# Supplementary material for: Propagation of seminal toxins through binary expression gene drives could suppress populations
Source: Sci Rep. 2022 Apr 15;12:6332. doi: 10.1038/s41598-022-10327-4 (PMC9012762; doi:10.1038/s41598-022-10327-4)
Supplement: Supplementary file 1 — Supplementary Information. [file 41598_2022_10327_MOESM1_ESM.pdf]

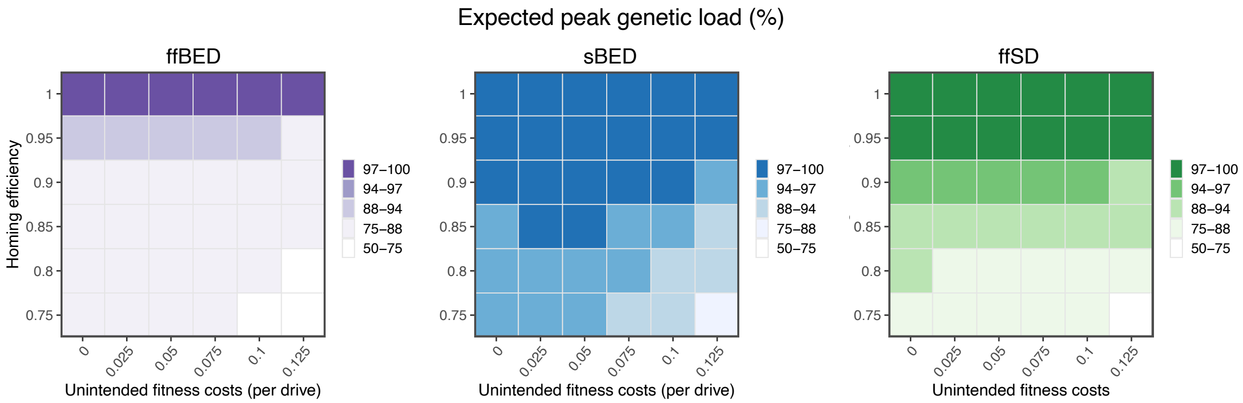

Fig. S1 Peak genetic load imposed by ffBED, sBED, and ffSD as a function of *Homing efficiency* and *Unintended fitness costs*. The expected peak genetic load was estimated, for each drive design, as the maximum averaged-across-simulations genetic load observed among adult genotypes within 36 generations.

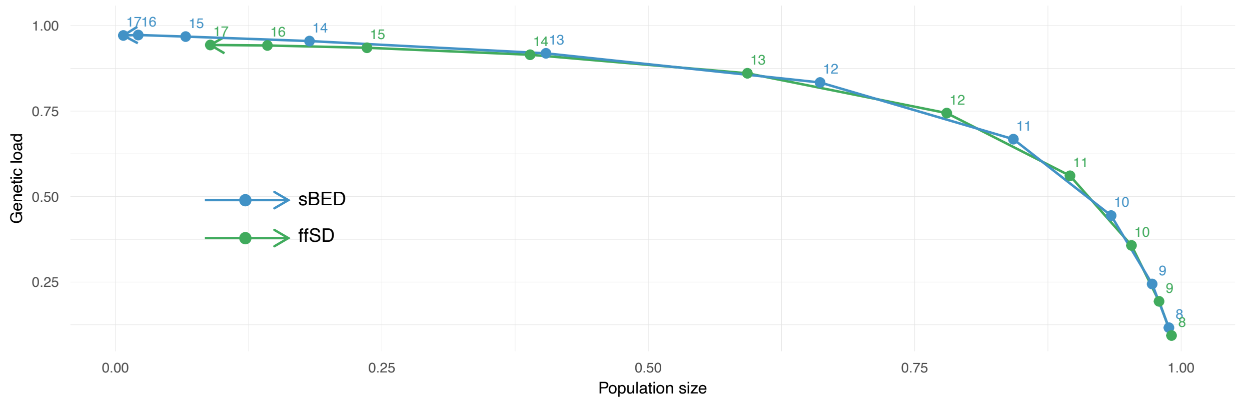

Fig. S2 Relationship between the observed genetic load and the relative population size across generations 8 to 17, where changes in population size mainly occur, for sBED and ffSD. Numbers stand for generations and points represent values averaged across the 100 performed simulations.

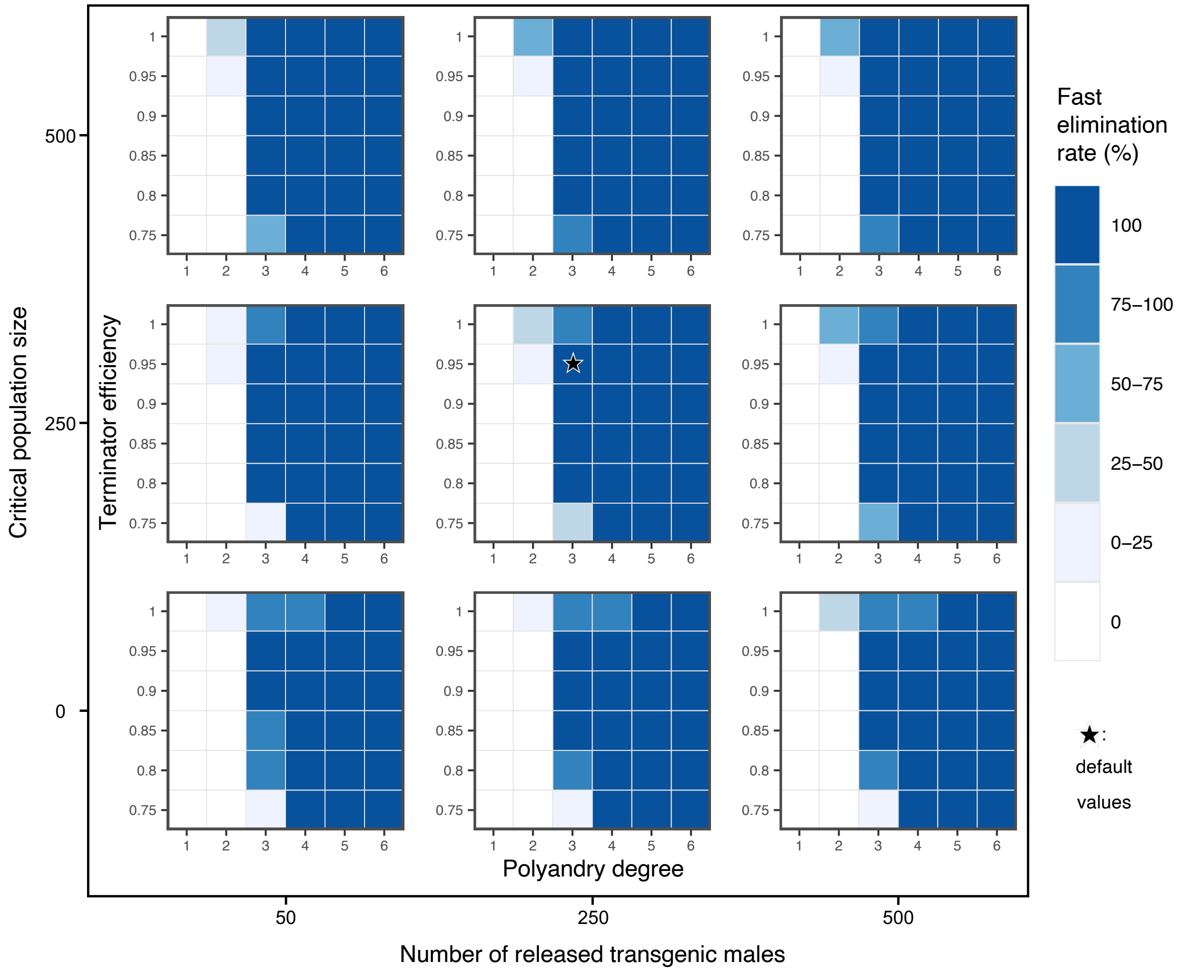

Fig. S3 Fast elimination rate of sBED for a combined space of multiple parameters. The percentage of simulations where the population was eliminated within 36 generations is shown for varying values of the following parameters: *Polyandry degree*, *Terminator efficiency*, *Release size* (the number of released males per drive), and *Critical population size*.

Table S1 Fast elimination rate of ffSD for a combined space of *Release size* (the number of released males per drive) and *Critical population size*.

Critical population size	Number of released transgenic males		
	100	500	1000
500	95%	99%	99%
250	91%	97%	97%
0	82%	85%	83%

Table S2 Versions of the implemented softwares.

Software	Version
R core	4.0.0
ggplot2	3.3.5
logger	0.2.0
bettermc	1.1.1
dplyr	1.0.7
rlist	0.4.6.1
zeallot	0.1.0
stringr	1.4.0
